# Prevalence of periodontitis and alveolar bone loss in a patient population at Harvard School of Dental Medicine

**DOI:** 10.1186/s12903-019-0925-z

**Published:** 2019-11-21

**Authors:** Mohammad F. Helmi, Hui Huang, J. Max Goodson, Hatice Hasturk, Mary Tavares, Zuhair S. Natto

**Affiliations:** 1 Department of Periodontics and Community Dentistry, College of Dentistry, King Saud University, Riyadh, Saudi Arabia; 2 Department of Oral Health Policy and Epidemiology, School of Dental Medicine, Harvard University, Boston, MA USA; 3 Pre-doc student, School of Dental Medicine, Harvard University, Boston, MA USA; 4 Department of Oral Medicine, Infection, and Immunity, School of Dental Medicine, Harvard University, Boston, MA USA; 5 Department of Applied Oral Sciences, Center for Clinical and Translational Research, The Forsyth Institute, Cambridge, MA USA; 6 Department of Dental Public Health, Faculty of Dentistry, King Abdulaziz University, Jeddah, Saudi Arabia

**Keywords:** Systemic diseases, Periodontal disease, Alveolar bone loss, Prevalence

## Abstract

**Background:**

Although several studies assessed the prevalence of alveolar bone loss, the association with several risk factors has not been fully investigated. The aim of this article is to measure the prevalence of periodontitis by calculating the mean alveolar bone loss/level of posterior teeth using bitewing radiographs among the patients enrolled in the clinics at Harvard School of Dental Medicine and address risk factors associated with the disease.

**Methods:**

One thousand one hundred thirty-one patients were selected for radiographic analysis to calculate the mean alveolar bone loss/level by measuring the distance between the cementoenamel junction and the alveolar bone crest on the mesial and distal surfaces of posterior teeth. Linear regression with Multi-level mixed-effect model was used for statistical analysis adjusting for age, sex, race, median household income, and other variables.

**Results:**

Mean alveolar bone level of the whole sample was 1.30 mm (±0.006). Overall periodontitis prevalence for the sample was 55.5% (±1.4%). Moderate periodontitis prevalence was 20.7% (±1.2%), while 2.8% (±0.5%) of the whole sample had severe periodontitis. Adjusted mean alveolar bone loss was higher in older age groups, males, Asian race group, ever smokers, and patients with low median household income.

**Conclusion:**

The effect of high household income on the amount of bone loss can be powerful to the degree that high household income can influence outcomes even for individuals who had higher risks of developing the disease. Public health professionals and clinicians need to collaborate with policy makers to achieve and sustain high quality of healthcare for everyone.

## Background

Periodontal diseases are inflammatory diseases of the oral cavity that can be confined only to the gingiva as in gingivitis or exceed beyond that to result in soft and hard tissue loss which affects the attachment of the teeth to the alveolar bone, as in periodontitis [1]. This is an inflammatory process that has been studied for decades with respect to its nature, risk factors, and whether it has specific or non-specific etiological factors including the underlying microbiology [2–5]. It is commonly found as a chronic state of disease that is characterized by slow bursts of progression of varying durations [6–9]. However, other studies are still investigating the nature of periodontal diseases progression. Emerging evidence suggesting that both theories of progression, linear and burst theories, are manifestations of the same phenomena and occur simultaneously in the same patients [10, 11]. Research continues to define all the factors participating in the initiation and progression of periodontal diseases. Cekici et al. published a report in 2015 discussing this particular inflammatory process and the mechanisms behind its occurrence [12]. The authors concluded, “*… Physiologic inflammation is a well-orchestrated network of cells, mediators and tissues. It is very important to consider the inflammatory / immune response as a whole, rather than many different modules working separately. As disease appears to be the result of loss of regulation and a failure to return to homeostasis, it is important to achieve a more complete understanding of the molecular and cellular events in this complex system*”. Overall, periodontal diseases have common etiological factors and many risk factors predisposing disease initiation and progression [13, 14]. Several studies found that although periodontitis occurs in most age groups, it is more prevalent in older age groups and seniors [15–17]. Ethnicity and race also play a role in the individuals’ susceptibility to periodontitis, with African Americans as more susceptible than other racial groups and ethnicities [18]. Some investigators have suggested that Mexican-Americans have the highest susceptibility to periodontal attachment loss [19]. Many studies have found that men have greater risk than women for advancing periodontal diseases [15, 16]. Several studies have also reported that individuals with lower socioeconomic status are at greater risk of periodontitis [15, 16].

Other risk factors reported to be associated with periodontitis include oral hygiene status [20, 21], smoking [22], and systemic diseases, particularly diabetes mellitus and cardiovascular diseases [22–24]. Evidence suggests that periodontitis and diabetes mellitus have two-way relationship with diabetes increasing the risk for periodontitis, and periodontal inflammation affecting the glycemic control in a negative way [25]. Debates continue on the nature of the relationship between systemic and periodontal diseases. In fact, it is still unclear in the literature including the investigators several aspects such as which came first, the pathogenesis, and treatment. Lewis et al., in 2017, published a review discussing the relationship between a number of systemic diseases and periodontitis and concluded that confounding prevents solid inference [26].

The National Institute of Dental and Craniofacial Research refers to periodontal diseases as the most common cause of tooth loss in adults [27]. In 2013, Marcenes et al. published a paper estimating the global burden of oral conditions from 1990 to 2010 [28]. In this paper, the disability adjusted life-years (DALYs) were measured, which is the sum of life years lost due to premature death and years lived with disabilities [29]. Based on this study, the global burden of oral conditions in 2010 affected nearly 4 billion people. Untreated dental caries of permanent dentition was ranked the first most prevalent condition affecting around 35% of all humans. Severe periodontitis was ranked the sixth most prevalent condition affecting about 744 million individuals globally. Over this twenty-year period, DALYs due to severe periodontitis had the highest increase of all oral conditions by 57%. Moreover, severe periodontitis is considered as the primary cause of DALYs in the age group of 35 to 59 year-old and accounted for more than five million DALYs globally, implying an average of 108 healthy life years per 100,000 people lost due to a preventable disease such as severe periodontitis. Such a high global burden of periodontal disease can have an undeniable negative impact on health, productivity, income, and overall quality of life.

Several studies have reported the prevalence of periodontitis in the United States. In 2015, Eke et al. published a paper using NHANES data from 2009 to 2012 finding that 46% of adults 30 years of age or older have periodontitis representing almost 65 million people with 9% having severe periodontitis [16]. A second paper from this group was published in 2016 measuring the prevalence of periodontitis for seniors 65 years of age or older [17]. The overall prevalence of periodontitis was 66% for all seniors 65 years of age or older with males to be more significantly affected by severe periodontitis (16%) compared to females (6%).

The use of radiographs to assess alveolar bone loss appears frequently in the literature [18]. The rational for using bitewing (BW) radiographs is to minimize angular distortion. Only in BW films does the x-ray beam penetrate perpendicularly through the teeth to the x-ray film or sensor while at the same time being parallel to the occlusal plane. An ideal bitewing radiograph should provide a clear view of the mandibular and maxillary alveolar bone and teeth with minimal overlap of anatomical structures [30]. Radiographic beam angulation has been reported to affect the radiographic measurements by an amount of ±1.6 mm comparing clinical and radiographic alveolar bone crest [31, 32]. The use of non-standardized BW radiographs, however, was reported in the literature to have the ability to detect less than 1 mm alveolar bone change indicating its usefulness for monitoring periodontal diseases progression [30, 33]. Studies that have used repeated radiograph measurements of the same sites have found a mean difference of 0.09 mm between the measurements suggesting a 9% discrepancy for repeated radiographs [34]. The specific aim of this study was to measure the prevalence of periodontitis using BW radiographs among the patients enrolled in the clinics at Harvard School of Dental Medicine (HSDM) and investigate their risk factors associated with the disease, comparing them to predisposing factors reported in the literature.

## Methods

The information technology (IT) team of HSDM performed a database search of up to 6265 patient records. The database search included completed comprehensive oral examinations and radiographs (either full mouth series or bitewing radiographs) for each individual patient who had a clinic visit between January 1, 2014 and December 31, 2015. Data were gathered from AxiUm®, an electronic health records system at HSDM, including age, gender, body mass index (BMI), chronic medical conditions (diabetes, hypertension, cardiovascular diseases, etc.), tobacco use, race/ethnicity, as well as each patient’s radiographs. The electronic health records did not contain information directly related to socioeconomic status (SES). To estimate SES we collected ZIP codes of all patients. Median income for each zip code was determined using U.S. Census Bureau statistics [35]. The patient pool was selected based on their age at their last appointment at HSDM. One examiner (MH) reviewed all 6265 patients and selected 2320 suitable patients for the study.

Exclusion criteria used were: 1) patients that were not within the specified age range, 2) patients with no BW radiographs, 3) patients with radiographs in which the cement-enamel junction (CEJ) and alveolar bone crest were not visible, 4) patients who did not have at least 2 approximating teeth or where the interproximal space was too narrow to observe the bone crest. Teeth were excluded if dental restorations obliterated the CEJ, rendering the distance between CEJ and alveolar crest questionable. Additionally, cases in which a tooth was found adjacent to an edentulous site with alveolar bone levels greater than 2 mm from the CEJ were not considered pathological due to possible surgical trauma. Any records indicating sites receiving osseous surgery or bone grafts were excluded. Third molar teeth were not included due to their tendency of not being captured by BW radiographs. Non-functional teeth were excluded for the possibility of super eruption. Alveolar bone loss/level was measured on the mesial and distal sites of first and second mandibular and maxillary posterior teeth using the calibrated measuring tool of Emago® (Oral Diagnostic Systems, Amsterdam, Netherlands) software– the radiographic imaging software at HSDM. Data will be available upon request. IRB approval was obtained through The Office of Human Research Administration, Harvard Faculty of Medicine, [45 CFR 46.101(b) [4]], Protocol # IRB16–1838. The permission from the HDSM to access AxiUm was included in the ethical approval.

### Outcome assessment

Radiographic indication of interproximal bone loss occurs when the distance between the CEJ and the alveolar bone crest is greater than or equal to 2 mm, as determined on a bitewing radiograph [36–39]. Our primary outcome is the level of alveolar bone on mesial and distal sites of posterior teeth as a continuous variable. We also categorized amount of bone loss based on case definition by the American Academy of Periodontology (AAP) into mild, moderate, and severe periodontitis [39–41] to estimate the prevalence of each case definition for descriptive and baseline characteristics.

### Predictors

Age: five categories of age were generated. Age groups of this study were defined as less than 30, 30–34, 35–49, 50–64, and 65 or more years old based on distribution of patients. Sex: binary variable of sex was coded 1 if the subject was male and 0 if subject was female. Race / Ethnicity: we generated 5 categories of the race variable based on patient self-reports. Categories included White, African American, Asian, Other, and Unknown. Since this was self reported, we did not have information about races that were reported as “Other”. Patients with Hispanic ethnicity were few (*N* = 21); hence it was coded under “Other”. BMI: based on patients’ reported height and weight, BMI was calculated and then categorized into 4 groups using CDC criteria [42]. Systemic Diseases: medical history of three main systemic diseases was collected from the electronic health records which were mandatory to fill. These included cardiovascular disease (CVD), hypertension, and type 2 diabetes. CVD and diabetes were coded 1 if the patient had the diseases and 0 if they did not. For hypertension, we relied on systolic and diastolic blood pressure measured at the clinic and not on medical history reporting hypertension because the later had a high number of misclassification resulting in very few records that were consistent in reporting the disease. We used the new categories by the American College of Cardiology and the American Heart Association [43] to develop 4 categories– normal, elevated, stage 1 hypertension, and stage 2 hypertension. Smoking status: we generated 3 categories to describe smoking status. Patients that did not smoke were categorized as never smokers, and patients who reported that they were smoking were categorized as current smokers. Patients who reported that they were smokers and had quit smoking were categorized as former smokers. Median household income: Since income was not included in the patient database, we based this variable on the ZIP code for each patient and the associated estimates of household income that have been collected using U.S. Census Bureau, 2012–2016 American Community Survey 5-Year Estimates [35]. The variable was categorized into either higher than the sample median household income (=1) OR equal or lower than the sample median household income (reference = 0).

### Sample size

To determine the adequate sample size, a power calculation was conducted. Based on a study conducted by Eke et al. in 2012, using similar age groups as the ones we used, the odds ratio of developing periodontitis was 2.315 comparing 35–49 year-old group to 30–34 year-old group, with a prevalence of total periodontitis of 36.6% [19], a sample size of *n* = 450 was adequate to obtain a Type I error rate of 5% and power greater than 80%. Based on the sample size calculations, each age group required a minimum of 90 patients; however, we conducted a random sample of 1300 out of the 2320 patients that were suitable for analysis and exceeded the minimum number of patients in each age category because we needed higher number of subjects in other categorical variables included. During the measurement process for all 1300 patients, 171 patients were excluded either due to closed electronic files or because their BW radiographs could not be calibrated with the measuring tool. The final number of patients that were included in the analysis was 1131 patients.

### Data analysis

**Fig. 1:**
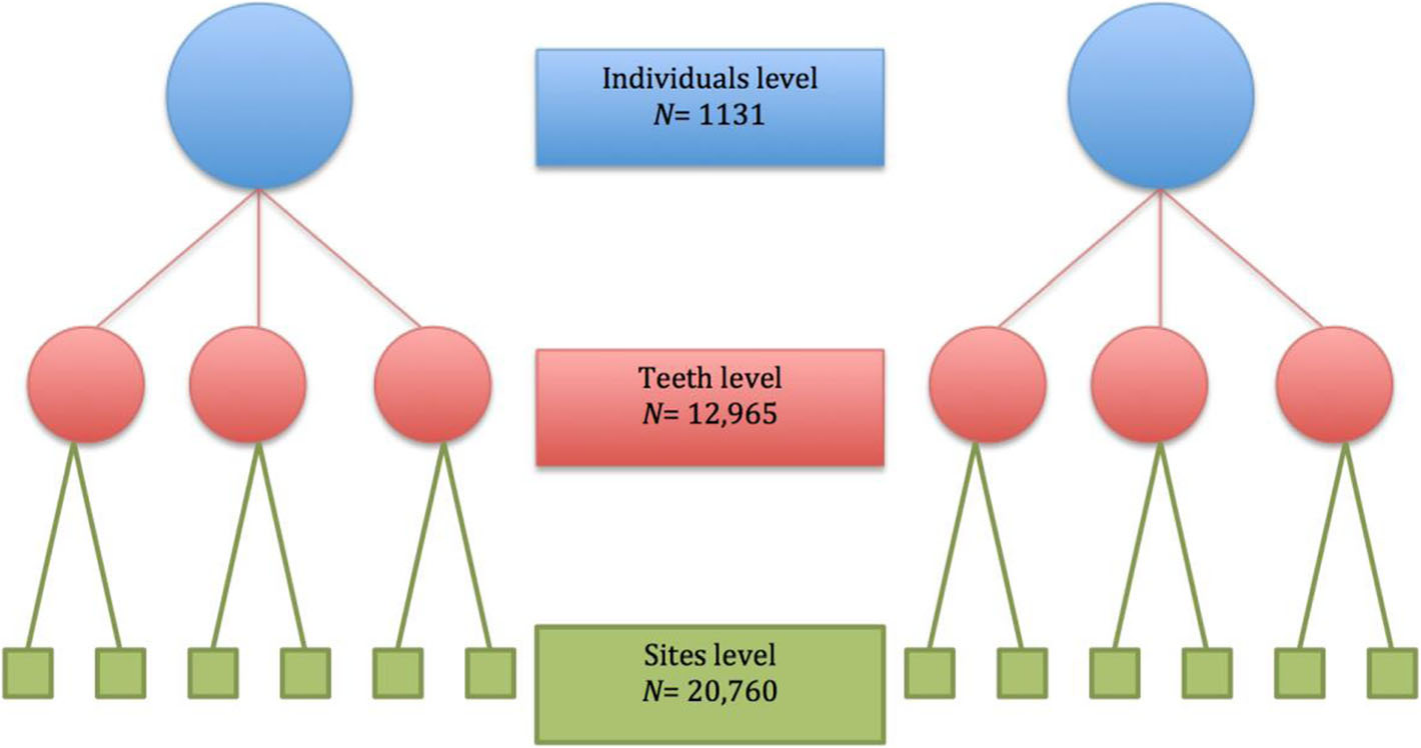
Multi-level Mixed-effect model

Descriptive statistics (means and standard deviations for continuous variables, counts and percentages for categorical variables) were calculated. The percentage of subjects with periodontal bone loss was computed for each age bracket. Statistical significance of the association between age and other predictors and periodontal bone loss was assessed via multi-level liner regression using mixed-effect model to estimate fixed-effect shared by the whole population and random-effect to account for variability between individuals and teeth examined. Sites measured are nested within teeth that are nested within individuals. Fixed-effect model estimates the grand mean of the population (intercept), and random-effect model estimates the standard deviation (variability) of each observation from its nested mean. Estimates of mixed-effect model explained more in Table 1 and Fig. 1. *P*-values less than 0.05 were considered statistically significant. All analyses were conducted using STATA V.14.2 statistical software package.

**Table 1:** Multi-level Mixed-effect model estimates

Fixed-effect Model	Intercept (grand mean) for whole sample
Random-effect Model	Allows variability between individuals, teeth, and sites
SD Individuals	SD of each individual’s mean from the overall grand mean
SD Teeth	SD of each tooth’s mean from its individual’s mean
SD Sites	SD of each observation (site) from its tooth’s mean

### Intra- and inter-examiners calibration

Inter and intra examiner reliabilities were conducted for the 2 examiners in this study. We were able to exceed the number of calibrations recommended by Fleiss [44] and the 2 examiners assessed BW radiographs for 80 randomly selected patients, measuring the alveolar bone level from the most coronal point of the crestal bone to the most apical point of the CEJ on the mesial and distal surfaces of posterior teeth with the measuring ruler being parallel to the root of each surface. The measurements were repeated one week later with the initial reading being blinded. To achieve a high consistency, each examiner repeated the measurements until we achieved a high degree of agreement between the readings of the same set. Further, inter-examiner reliability test was conducted to eliminate the possibility of chance agreement. Two-way random-effects [45] intra class correlation coefficient (ICC) test was performed to check both intra- and inter-examiner reliabilities using STATA V.14.2 statistical software. ICC agreement is interpreted as poor if it scores less than 0.40, fair between 0.40 to 0.59, good between 0.60 to 0.74, and excellent if it scores between 0.75 and 1.00 [46].

### Radiographic discrepancy

We wanted to measure the expected magnification discrepancy of the x-ray machines that were used to take the radiographs of our sample of interest. A random sample of 22 BW radiographs were selected and measured for the widths of the implants then compared to the true measurements provided by the clinician in the patients’ medical records. The mean of radiographic measures was 4.5 (±0.47) while the mean of real measurements provided by the manufacture was 4.36 (±0.49).

We expected an amount of almost 15% magnification error that would affect our measurements. This magnification error was taken into account for generating variables based on periodontal disease case definition. Based on the AAP Task Force Report 2015, the earliest sign of mild periodontitis on radiographs is to observe bone loss (measured from CEJ to crestal bone) that is equal to or greater than 2 mm and less than or equal to 3 mm without any recommendations about magnification error correction [39].

**Fig. 2:**
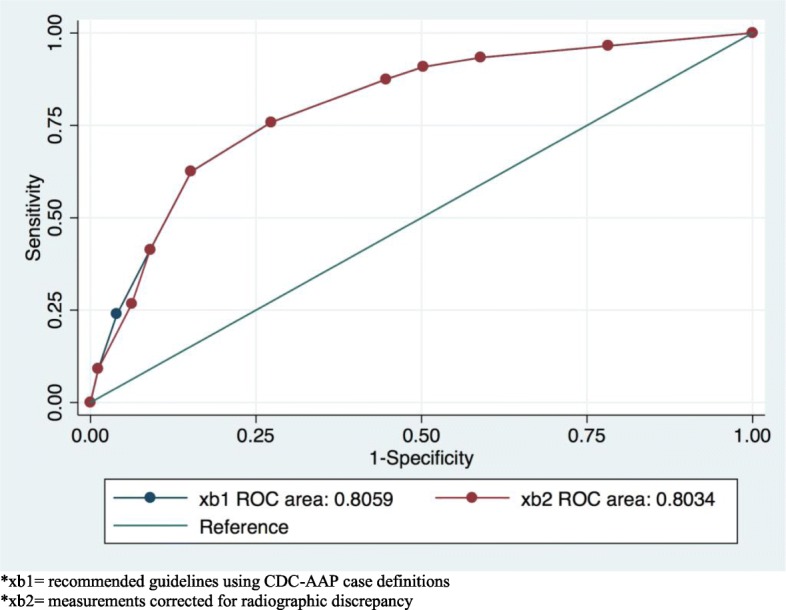
ROC curve of comparing CDC-AAP case definitions of periodontitis to 15% corrected measurements for radiographic magnification. *xb1 = recommended guidelines using CDC-AAP case definitions. *xb2 = measurements corrected for radiographic discrepancy

We adjusted for this error by incorporating 15% for every 1 mm in case definition variables generated. For instance, if a real measurement from CEJ to crestal bone is 1.8 mm, which is not an indication of mild periodontitis, the radiograph measurement for that site is expected to be 1.8 × 1.15 = 2.07 mm which might lead to overestimation of the diseases if we used 2 mm as the cutoff. Hence, we generated case definitions of periodontitis based on this expected radiography magnification error and for the example mentioned, a cutoff of 2.3 mm (corrected for radiographic magnification error) would not result in overestimation of the disease. We also generated periodontitis case definition based on the recommendation by AAP Task Force Report 2015 and compared the two cut-offs for sensitivity and false positive rate (Fig. 2). The two cut-offs did not significantly differ.

## Results

Table 2 presents results and randomly selected teeth for calibration testing.

**Table 2:** Intra and inter examiner reliability

Examiner	Site/Tooth	ICC Individual (95% CI)	ICC Average (95% CI)	*P* value
Intra M	M3	0.92 (0.87–0.95)	0.96 (0.93–0.97)	*P* < 0.0001
Intra H	M3	0.92 (0.86–0.95)	0.95 (0.92–0.97)	*P* < 0.0001
Inter M & H	M3	0.80 (0.68–0.87)	0.88 (0.81–0.93)	*P* < 0.0001
Inter M & H	D13	0.95 (0.92–0.97)	0.97 (0.95–0.98)	*P* < 0.0001

*N* = 80 subjects. Examiner 1 (M), had 0.92 agreement (95% CI 0.87–0.95) for repeated individual measurements and 0.96 (95% CI 0.93–0.97) for averages agreement for intra-examiner reliability comparing the first and second times measurements of alveolar bone level for the mesial site of tooth number 3 for the same subset. Examiner 2 (H), had an agreement of 0.92 (95% CI 0.86–0.95) for individual measurements and 0.96 (95% CI 0.92–0.97) for averages agreement for the same variable. We also had excellent agreement for inter-examiner reliability between the two examiners. For example, consistency of agreement for inter-examiner reliability comparing the alveolar bone level measurement on the mesial site of tooth number 3 is 0.80 (95% CI 0.68–0.87) for individual measurements and 0.88 (95% CI 0.81–0.93) for average agreement for the same subset. Another example of higher agreement is shown for the distal side of tooth number 13

### Descriptive statistics

A total of 1131 individuals were included in the analysis with a mean alveolar bone level of 1.30 mm (±0.006). Mean bone level ranged between 0.77 mm (±0.006) for the youngest group to 2.04 mm (±0.019) for the oldest. 55% of the sample was composed of females with a mean bone level of 1.26 mm (±0.008) compared to 45% males with a mean bone level of 1.34 (±0.009). White race composed 36.5% of the sample, followed by Other (22.1%), Unknown (20.5%), African American (9.0%), and Asian (7.5%). Table 3 presents descriptive statistics for the whole sample.

**Table 3:** Prevalence of mild, moderate, and severe periodontitis among different groups of patients visiting HSDM

Percentage (%)									
	*N (%)*	Mild	SE	Moderate	SE	Severe	SE	MABL* (mm)	SE
Total	1131 (100.0)	55.5	1.4	20.7	1.2	2.8	0.5	1.30	0.006
Age Groups (yrs)									
< 30	247 (21.8)	17.0	2.3	1.2	0.7	0.0	n/a	0.77	0.006
30–34	108 (9.5)	33.3	4.5	3.7	1.8	0.9	0.9	0.90	0.012
35–49	305 (27.0)	51.8	2.8	12.7	1.9	1.9	0.8	1.24	0.010
50–64	300 (26.5)	80.6	2.2	37.3	2.8	4.3	1.1	1.80	0.013
65+	171 (15.1)	86.5	2.6	44.5	3.8	7.0	1.9	2.04	0.019
Gender									
Male	508 (44.9)	57.0	2.2	24.1	1.9	4.3	0.9	1.34	0.009
Female	623 (55.1)	54.1	1.9	18.0	1.5	1.6	0.5	1.26	0.008
Race									
White	413 (36.5)	56.1	2.4	20.3	1.9	2.6	0.7	1.30	0.009
African American	100 (8.8)	53.0	5.0	27.0	4.4	9.0	2.8	1.36	0.028
Asian	85 (7.5)	62.3	5.2	29.4	4.9	1.1	1.1	1.43	0.020
Other	250 (22.1)	58.0	3.1	20.0	2.5	1.6	0.7	1.29	0.012
Unknown	283 (25.0)	48.2	3.2	15.1	2.3	3.0	1.1	1.24	0.013
Median Household Income									
Lower than median	510 (45.1)	55.1	2.2	22.1	1.8	3.9	0.8	1.32	0.009
Higher than median	621 (54.9)	55.5	1.9	19.4	1.6	1.9	0.5	1.28	0.007
Body Mass Index									
Underweight	27 (2.4)	18.5	7.6	11.1	6.1	0.0	n/a	0.92	0.031
Normal	413 (36.5)	52.5	2.4	19.1	1.9	2.6	0.8	1.23	0.009
Overweight	263 (23.2)	60.8	3.0	23.2	2.6	2.2	0.9	1.40	0.013
Obese	137 (12.1)	59.1	4.2	23.4	3.6	3.6	1.6	1.45	0.021
Not reported	291 (25.7)	56.0	2.9	20.3	2.3	3.4	1.0	1.30	0.011
Smoking Status									
Never smoker	668 (59.0)	49.5	1.9	15.8	1.4	2.3	0.6	1.19	0.007
Former smoker	141 (12.4)	70.2	3.8	41.1	4.1	4.2	1.7	1.72	0.021
Current Smoker	82 (7.2)	58.5	5.4	19.5	4.4	2.4	1.70	1.41	0.023
Not Reported	240 (21.2)	61.6	3.1	22.5	2.7	3.3	1.1	1.38	0.013
Diabetes									
Yes	60 (5.3)	75.0	5.6	40.0	6.3	6.7	3.2	1.81	0.038
No	1071 (94.7)	54.2	1.5	19.6	1.2	2.6	0.5	1.28	0.006
CVD									
Yes	132 (11.7)	79.5	3.5	32.6	4.1	3.0	1.5	1.66	0.020
No	999 (88.3)	52.1	1.5	19.1	1.2	2.8	0.5	1.26	0.006
Hypertension									
Normal	346 (30.6)	46.8	2.6	15.6	1.9	1.4	0.6	1.18	0.009
Elevated	157 (13.9)	54.1	3.9	21.6	3.2	3.8	1.5	1.35	0.016
Stage 1	281 (24.9)	59.7	2.9	24.5	2.5	2.1	0.8	1.37	0.012
Stage 2	125 (11.0)	70.4	4.1	31.2	4.1	8.0	2.4	1.61	0.023
Not reported	222 (19.6)	55.4	3.3	17.1	2.5	2.2	0.9	1.23	0.013

*N* = 1131 patients (20,760 sites from 12,965 teeth)

*MABL Mean alveolar bone “level” in millimeter

### Severity of the disease and proportions of case definitions

**Fig. 3:**
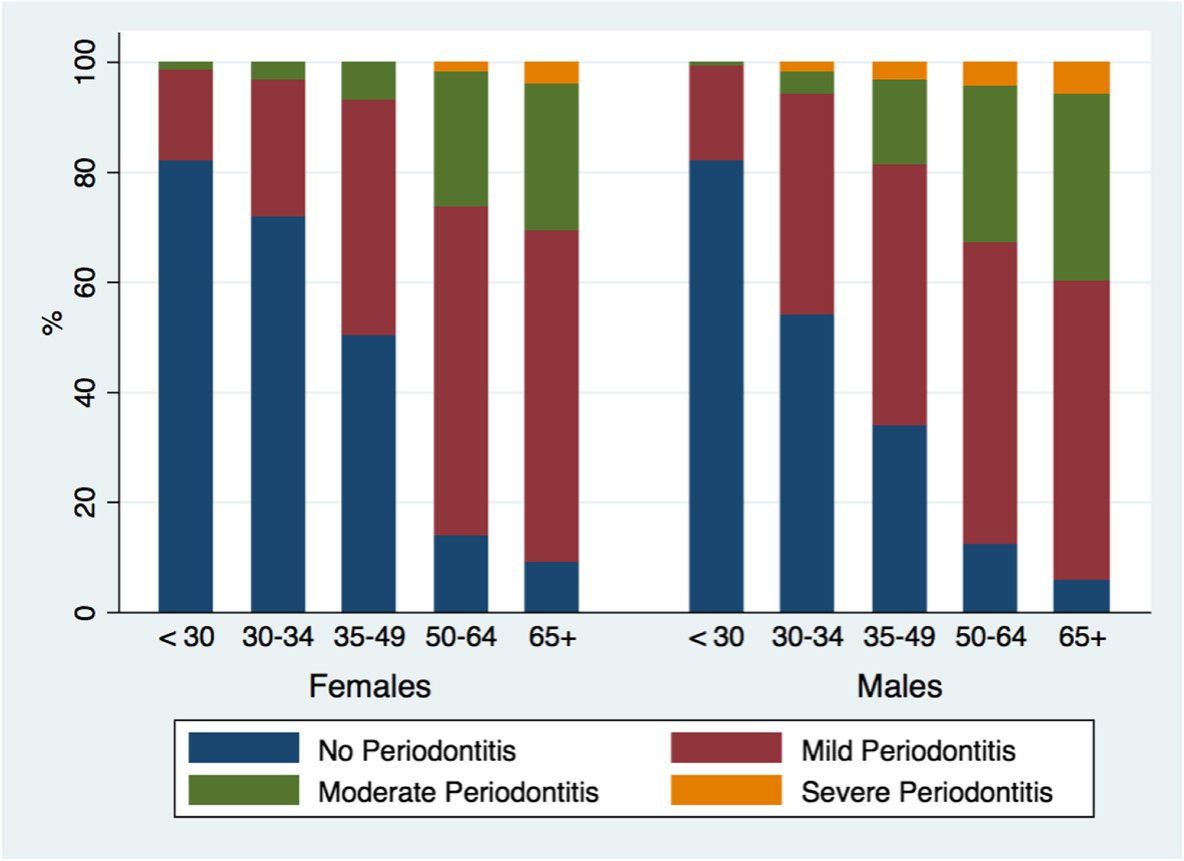
Prevalence of mild, moderate, and severe periodontitis over different age groups and gender

Overall periodontitis prevalence for the sample was 55.5% (±1.4%). Moderate periodontitis prevalence was 20.7% (±1.2%), while 2.8% (±0.5%) of the whole sample had severe periodontitis. All three case definitions were highest among 65+ year-old, males, former smokers, having CVD, and stage 2 hypertension subjects. More detailed prevalence of each case definition across different groups is also presented in Table 3. Furthermore, Fig. 3 illustrates prevalence of mild, moderate, and severe periodontitis across different age groups and genders.

### Adjusted estimates (multi-variable analysis)

All variables included in the multi-variable model had significant associations with the outcome except for African American race, diabetes, CVD, and hypertension (they were significant in bivariate analyses). Mean increase in bone loss was higher for older age groups compared to younger age groups with 65+ year-old exhibiting the highest amount of bone loss, adjusting for sex, race, household income, BMI, smoking, reported CVD, diabetes, and hypertension. Males had higher amounts of bone loss than females (Mean difference = 0.096 mm [95% CI: 0.04, 0.14. *P*-value < 0.001]), Asian race had higher bone loss compared to White race (Mean difference = 0.23 mm [95% CI: 0.13, 0.33. *P*-value < 0.001]), and higher household income was also associated with reduced amount of bone loss compared to lower income (Mean difference = − 0.06 mm [95% CI: − 0.11, − 0.007. P-value < 0.026]). For BMI, the association was not significant for any categories except for the obese group which showed a significant decline in bone loss compared to the normal weight group equal to − 0.13 mm (95% CI: (− 0.22)-(− 0.04). *P*-value = 0.003). We introduced interaction terms to assess any effect measure modification between BMI and other covariates. We did not find any significant interactions except for median household income and BMI. It showed a decreased amount of bone loss for obese subjects who also had higher than median household income (Mean difference = − 0.25 mm [95% CI: − 0.38, − 0.12. *P*-value < 0.001]). Compared to never smokers (reference group), current smokers had higher amounts of bone loss than former smokers. Table 4 provides details of each adjusted mean change in bone level (bone loss) for all variables included in the analyses. For the random effect part, we found that estimates (mean change), compared to their nested means, varied between individuals and teeth by 0.164 mm (95% CI: 0.15, 0.18) and 0.066 mm (95% CI: 0.060, 0.072), respectively. Random-effect coefficients are also provided in Table 4.

**Table 4:**
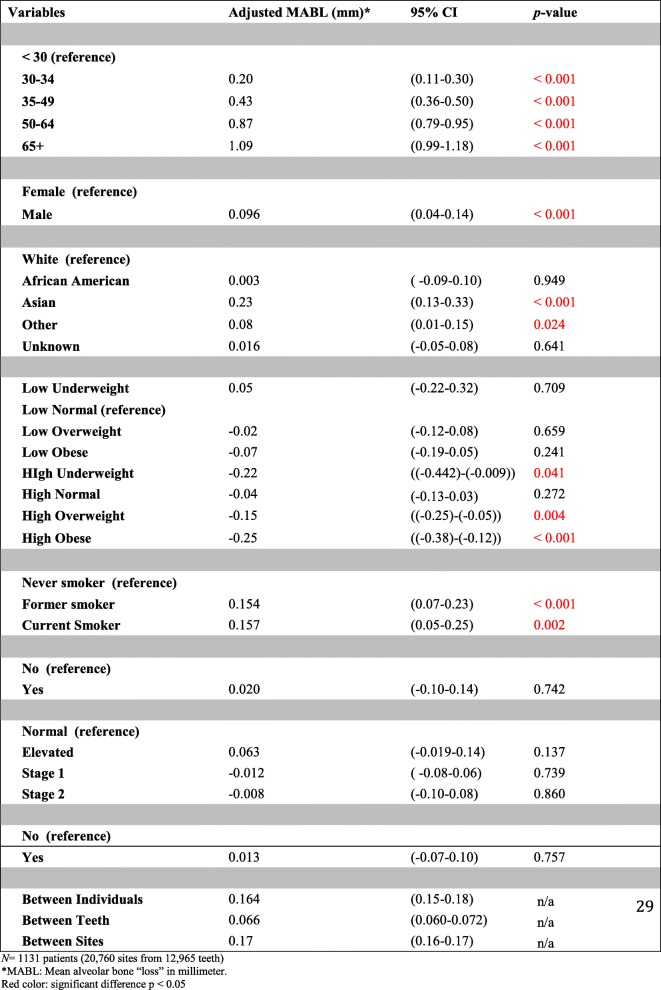
Adjusted mean alveolar bone loss (mm) with different predictors included in the model (multi-variable analysis)

*N* = 1131 patients (20,760 sites from 12,965 teeth)

^a^*MABL* Mean alveolar bone “loss” in millimeter

Red color: significant difference *p* < 0.05

## Discussion

Our results are in agreement with similar studies of prevalence of periodontal diseases among different groups that exhibit specific features and risk factors to periodontal diseases. This is the first time to use Harvard School of Dental Medicine records with this number of patients. A study conducted by Eke et al. in 2012 to evaluate the prevalence of periodontitis in adults in 2009–2010 showed, as in our study, that older age groups have a higher risk and proportion of periodontal diseases compared to younger age groups [19]. Our results indicate that males have a higher risk of developing periodontal diseases with significantly higher alveolar bone loss compared to females and this result coincides with similar results reported in literature indicating males having higher risk of developing the disease [15, 19, 47]. Many studies have reported that smoking is a primary predictor of periodontal diseases [14, 48–52]. In our study, current and former smokers had increased mean alveolar bone loss compared to never smokers (Table 4).

In our study, subjects with higher than median household income were associated with 0.06 mm lower rate of bone loss compared to subjects with lower than median income (95% CI: − 0.11, − 0.007, *p*-value = 0.026). Observations of higher risk to periodontal diseases to poverty and low household income have been reported in multiple studies in the literature [15, 53]. Our results also showed significant reduction in bone loss also for individuals who were categorized as obese by BMI. Limitations, however, exist for BMI, as a sole indicator for obesity; BMI measurements may be misleading because it is a measure for excess weight not excess body fat [54]. Hence, interpretation of BMI associated estimates to the outcome should be interpreted cautiously. We further analyzed this observation to check for any misclassification. Since median household income was the only variable that was associated with decreased risk of bone loss, we created different interaction terms between median income variable and different predictors. We found that subjects who were obese with higher than median household income had 0.25 mm lower rate of bone loss (95% CI: − 0.38, − 0.12. *p*-value< 0.001) compared to individuals who had normal weight and low income. This observation suggests that subjects with physiological risk factors would have better health and less adverse outcomes if they had had higher incomes. Social determinants of health may play a crucial role in this observation especially in its geographic, socioeconomic status, and educational backgrounds. Further analysis of social determinants of health is a necessity to understand all factors participating in the development of diseases and their associated risk factors.

One of the strengths of the study is helps to identify patient groups within a closed system (HSDM clinics) with higher oral health needs. Identifying those who come from areas with low median household income can play a key factor in planning treatment, offering patients more services, and, on a larger scale, searching for ways to enhance the access to healthcare system in those areas. Another strength of this study is that measuring protocol was carefully calibrated with an overall excellent inter- and intra-examiner reliability testing.

There are definite limitations, however, focusing on posterior teeth and using only BW radiographs in school records, is one way to conduct a partial mouth periodontal examination (PMPE). PMPE shows tendencies to underestimate prevalence of periodontal diseases when compared to full mouth periodontal examination (FMPE) [55, 56]. Furthermore, BW radiographs have a limitation in detecting craters, furcation involvements, and different angular defects [57–61] which would result in underestimating the prevalence of the diseases. More precise radiography techniques and periodontal diseases measuring protocols would overcome some of the limitations of this study. Another limitation is several factors should be considered in the future when we study the relationship between household income/systemic diseases and bone loss such as background characteristics, number of visits, oral hygiene and plaque index.

## Conclusions

Although limitations exist in our study, results of this study indicate that different predictive factors have different risks of the progression of periodontal diseases. Primary factors that were associated with higher rates of bone loss were older age, male, Asian racial group, and smoking. Moreover, low income could be one barrier for accessing healthcare systems, dental or medical in general, and thus may play an important role in determining the severity and prevalence of diseases. Our results show that individuals with high household income had lower prevalence of periodontal diseases and lower amount of bone loss compared to individuals with low household income. This manifestation of protective effect by high household income on the amount of bone loss can be powerful to the degree that income can influence the outcome even for individuals who had higher risk of developing the disease. Further analysis of social determinants of health is a necessity to understand all factors participating in the development of diseases and their associated risk factors. Public health professionals and clinicians need to collaborate with policy makers to achieve and sustain high quality of healthcare for everyone. Addressing and evaluating areas with low household income for further investigation is necessary to attain and sustain equality of access to the healthcare system.

## Data Availability

The dataset used during the study are available from the corresponding author upon request.

## Abbreviations

AAP: American Academy of Periodontology
BMI: Body mass index
BW: Bitewing
CEJ: Cement-enamel junction
CVD: Cardiovascular disease
HSDM: Harvard School of Dental Medicine
NHANES: National Health and Nutrition Examination Survey
SES: Socioeconomic status
